# Abscess Formation After Tooth Extraction: A Long-Term Complication of Polyacrylamide Hydrogel Filler

**DOI:** 10.7759/cureus.28180

**Published:** 2022-08-19

**Authors:** Aydan A Kose, Can Ekinci, Atacan E Koçman

**Affiliations:** 1 Plastic, Reconstructive and Esthetic Surgery, Osmangazi University School of Medicine, Eskişehir, TUR; 2 Plastic, Reconstructive and Esthetic Surgery, Private Practice, İstanbul, TUR

**Keywords:** filler complication, filler infection, permanent filler, abscess, paag, polyacrylamide hydrogel

## Abstract

A 35-year-old woman with a history of polyacrylamide hydrogel filler injection was referred with a fluctuating facial abscess after decayed tooth extraction. MRI imaging confirmed the diagnosis of an abscess. After appropriate treatment, the patient healed with a little hyperpigmentation and deformity in the zygomaticotemporal area. Although polyacrylamide hydrogel filler injection is considered non-toxic, non-immunogenic, and biocompatible; as a permanent material, physicians should be aware of the risk of its late complications such as late infections. In addition to antiseptic measures, antibiotic prophylaxis may be necessary before the procedures which have a risk of bacteremia and close to the permanent filler location.

## Introduction

The complication rate of filler injections can be as high as 18% [1]. Fortunately, most of the complications reported were resolved by some minor interventions or even spontaneously. However, even much more serious complications like anaphylactoid reactions, abscesses, or vascular problems like blindness or skin necrosis may be seen after filler injections [2-3]. In this article, we present a patient with an abscess formation several years after polyacrylamide hydrogel (PAAG) injection. The uniqueness of this case is that it is the first filler infection reported after a surgical intervention to our knowledge.

## Case presentation

A 35-year-old non-smoker, healthcare worker woman was admitted to the emergency department with a high fever, facial swelling, and difficulty in swallowing a day after decayed tooth extraction. There was no previous facial surgery or intervention history except for an injection of a facial filler five years ago. On the physical examination, prominent swelling and hyperemia were present on the left side of the face, buccal area, and the oropharynx. The laboratory findings showed a mild increase in acute phase reactants. The patient was hospitalized by the Department of Infectious Diseases and given parenteral antibiotherapy of ampicillin/sulbactam and ciprofloxacin.

**Figure 1:**
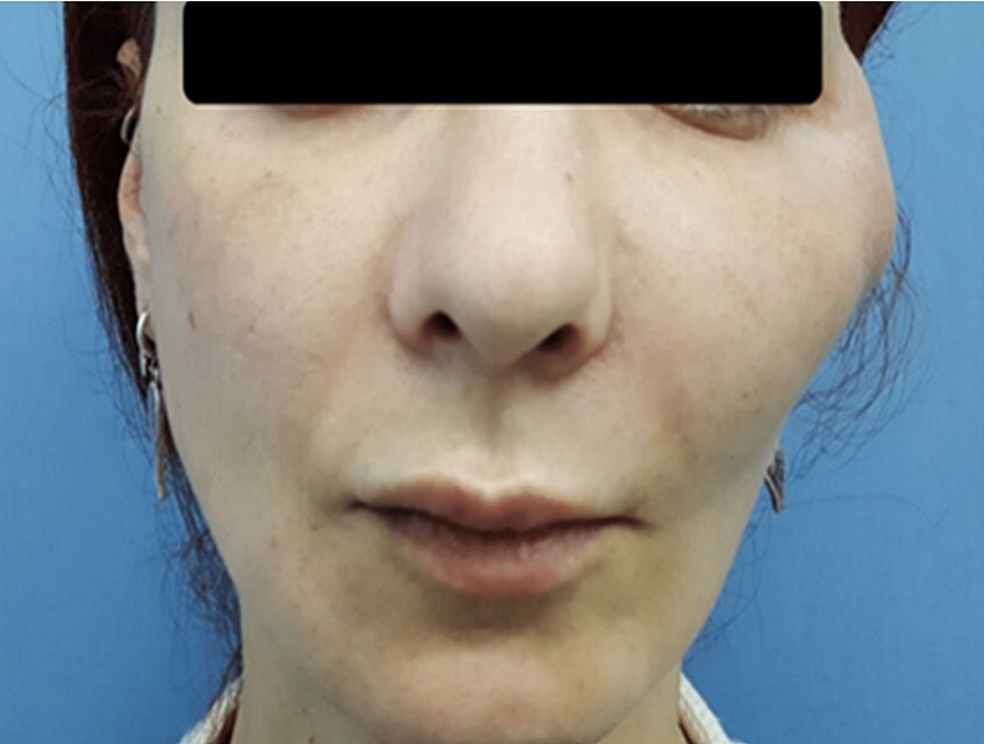
A localized fluctuating abscess with vascular circulatory impairment on the left zygomatic area.

**Figure 2:**
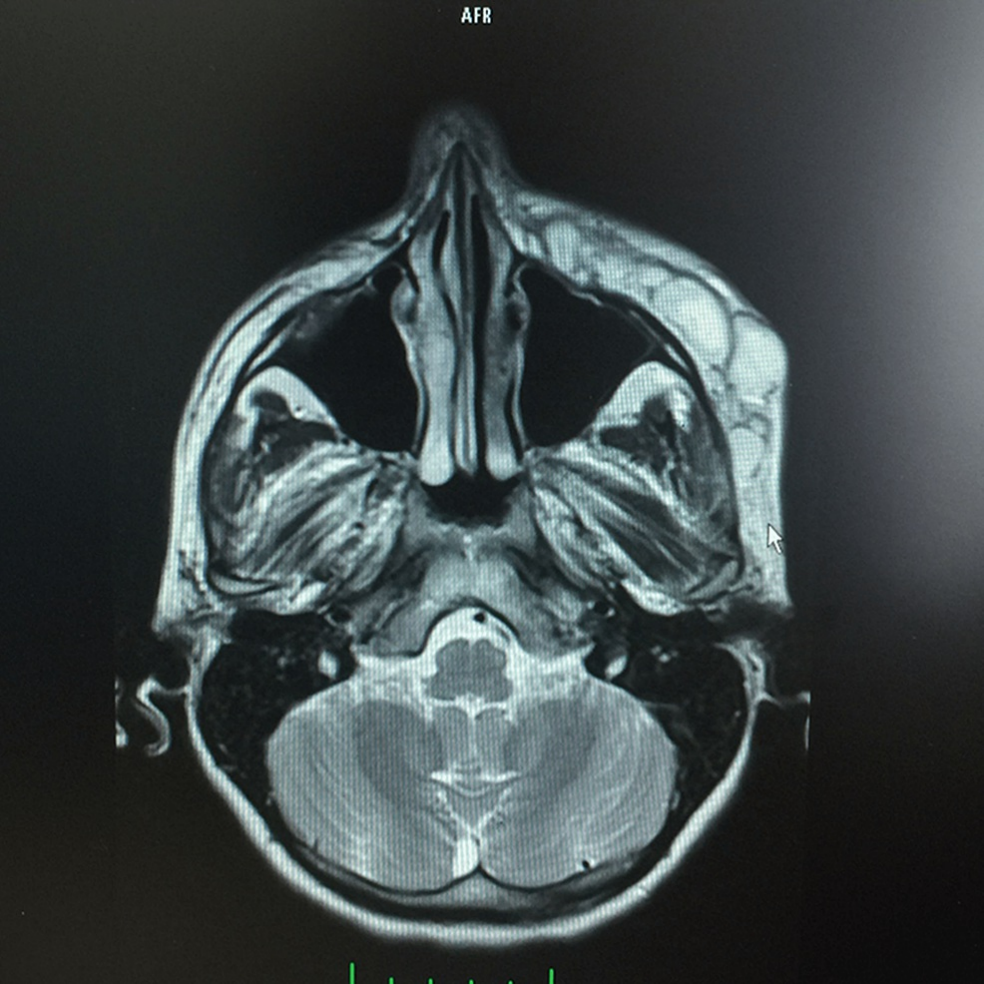
An MRI image showing multiloculated abscess on the left zygomatic arch and temporal bone.

**Figure 3:**
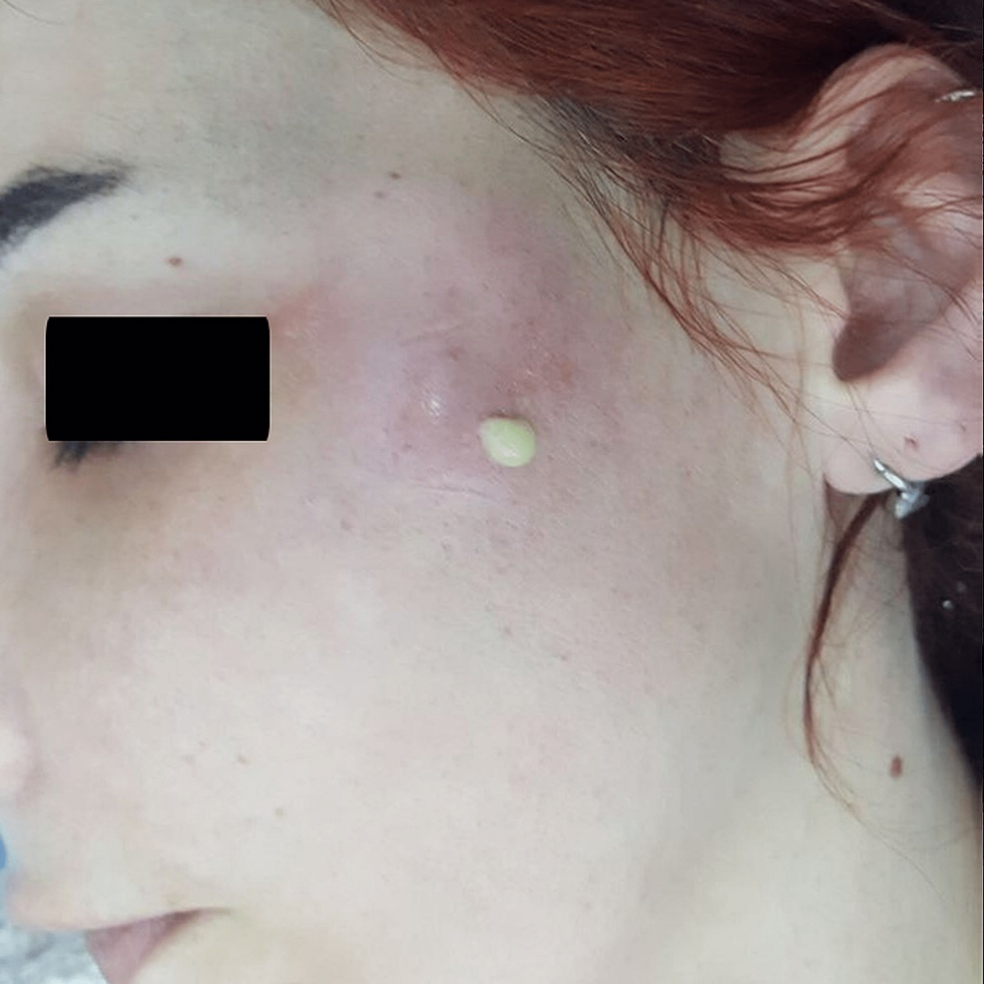
Drainage of the abscess and pus coming out.

During her hospitalization, the prominent edema on her face gradually regressed and a localized, fluctuating abscess causing circulation impairment of the skin above appeared on the fifth day (Figure 1). MRIs of the patient showed multiloculated abscess formation (Figure 2). She was consulted to us for the abscess drainage. The abscess has been drained and malodorous, yellowish pus leaked out (Figure 3). A swab sample was taken from the pus for culture.

With the detailed history, it was understood that she had PAAG filler injection over her bilateral cheek bone and temporal areas five years ago. During the follow-ups, there was no bacterial growth on the culture. After the treatment, the patient healed with some hyperpigmentation and deformity on the zygomatic area.

## Discussion

Among a wide variety of fillers, PAAG fillers are relatively new generation permanent fillers that are presented to the market as non-toxic, non-immunogenic, non-embryotoxic and biocompatible products. They have been in use for more than 25 years and got Conformité Européenne (CE) certification in 2001 [4]. 

It is composed of 2.5% polymer of acrylamide monomer and 97.5% non-pyrogenic water and is a jellylike colorless substance [5]. Its hydrophilic nature allows continuous water exchange between the hydrogel and the surrounding tissues, which is proposed to prevent biofilm formation. In comparison to other fillers with micro-particles, its filling effect does not rely on a foreign body reaction. Due to these characteristics and its hydrophilic property, PAAG is considered a very convenient filler in non-invasive rejuvenation procedures and is used widely all around the world [4].

Late infections of the PAAG filler material are rare compared to the other complications and they are usually caused by repeated injections to the previously treated sites or an accidental puncture of the injected site during supplement injections such as botulinum toxin, filler, or lipolysis [6]. In fact, careless antiseptic preparation before the injection may cause transmission of the epidermal flora into the filler giving rise to bacterial colonization. Christensen et al. hypothesized that many filler complications which were regarded to be caused by product-tissue incompatibility might be low-grade infections caused by dormant bacterial contamination de facto. They detected several Gram-positive, skin-associated bacteria by Gram staining, RNA sequencing, and fluorescent in situ hybridization (FISH) in the specimens of 59 patients with an adverse reaction to PAAG while the cultures were negative [7]. Cassuto and Sundaram also support this hypothesis and moot that many “so-called filler granulomas” are indeed inflammations; culture negativity should not exclude infection as many Mycobacterial abscesses or granulomas may present as sterile nodules [8]. Consistent with the literature, there was no bacterial growth on the culture in our case but unfortunately, Gram staining, RNA sequencing, or FISH was not performed. In short, bacteria isolation from the drainage material is uncommon with swab cultures in the literature. Therefore, direct microscopy or other techniques are strongly recommended to identify the source of the bacteria [9]. 

Unlike up-to-date literature, the starter of the infection in our case was a decayed and infected tooth extraction. Soon after extraction, a local infection caused an abscess formation in or around the PAAG filler. Systemic or local bacteremia during extraction of the infected tooth might have caused new colonization around or inside the filler or might have activated the dormant bacteria somehow which might have been colonized in the filler previously. The PAAG fillers do not incorporate with the living tissue but they are reported to be surrounded by a thin interlayer of connective tissue and elastic fibers [10], thus they can serve as a suitable medium for many bacteria species. Furthermore, since PAAG is not vascularized, immune access is limited which also facilitates bacteria to colonize easily. Therefore, local antiseptic measures or even prophylactic antibiotherapy might be necessary before the procedures carrying a bacteremia risk in patients with a history of PAAG filler injection.

## Conclusions

The uniqueness of this case is that it is the first filler infection reported after a surgical intervention to our knowledge. Any local procedure carrying a risk of bacteraemia may cause an inoculation of bacteria to the filler material or activate the dormant bacteria residing in the filler. Thereby, patients who have permanent fillers may need to use prophylactic antibiotics before surgical procedures which carry a risk of local or systemic bacteraemia.
